# Clinical Characteristics of Hospitalized Patients With COVID‐19 and Their Association With the Progression to Critical Illness and Death: A Single‐Center Retrospective Study From Northwestern Mexico

**DOI:** 10.1111/crj.13813

**Published:** 2024-07-16

**Authors:** Francisco A. Martínez‐Villa, Uriel A. Angulo‐Zamudio, Nidia Leon‐Sicairos, Ricardo González‐Esparza, Jaime Sanchez‐Cuen, Jesus J. Martinez‐Garcia, Hector Flores‐Villaseñor, Julio Medina‐Serrano, Adrian Canizalez‐Roman

**Affiliations:** ^1^ School of Medicine Autonomous University of Sinaloa Culiacan Sinaloa Mexico; ^2^ Departamento de Medicina general Unidad de Medicina Familiar No. 21, IMSS La Cruz de Elota Sinaloa Mexico; ^3^ Research Department Pediatric Hospital of Sinaloa Culiacan Sinaloa Mexico; ^4^ Research Department Hospital Regional, ISSSTE Culiacán Sinaloa Mexico; ^5^ Molecular Biology Department The Sinaloa State Public Health Laboratory, Secretariat of Health Culiacan Sinaloa Mexico; ^6^ Research Department Coordinación de Investigación en Salud, Delegacion IMSS Culiacan Sinaloa Mexico; ^7^ Research Department The Women's Hospital, Secretariat of Health Culiacan Sinaloa Mexico

**Keywords:** COVID‐19, critical illness, death, Mexico, SARS‐CoV‐2

## Abstract

**Objective:**

The objective of this study was to associate the epidemiological and clinical characteristics of patients hospitalized for COVID‐19 with the progression to critical illness and death in northwestern Mexico.

**Methods:**

From March to October 2020, we collected the demographic and clinical characteristics of 464 hospitalized patients from northwestern Mexico.

**Results:**

Sixty‐four percent (295/464) of the patients became critically ill. Age, occupation, steroid and antibiotic use at previous hospitalization, and underlying diseases (hypertension, obesity, and chronic kidney disease) were associated with critical illness or death (*p*: < 0.05). No symptoms were associated with critical illness. However, the parameters such as the heart rate, respiratory rate, oxygen saturation, and diastolic pressure and the laboratory parameters such as the glucose, creatinine, white line cells, hemoglobin, D‐dimer, and C‐reactive protein, among others, were associated with critical illness (*p*: < 0.05). Finally, advanced age, previous hospital treatment, and the presence of one or more underlying diseases were associated with critical illness and death (*p*: < 0.02).

**Conclusions:**

Several epidemiological (e.g., age and occupation) and clinical factors (e.g., previous treatment, underlying diseases, and vital signs and laboratory parameters) were associated with critical illness and death in patients hospitalized with COVID‐19. These data provide us with possible markers to avoid critical illness or death from COVID‐19 in our region.

## Graphical abstract

These data provide us with possible markers to avoid critical illness or death from COVID‐19. Heart rate, respiratory rate, oxygen saturation, and diastolic pressure were associated with critical illness by COVID‐19 in adults in northwestern Mexico. Laboratory parameters such as glucose, creatinine, white line cells, hemoglobin, D‐dimer, and C‐reactive protein, among others, were associated with critical illness. Advanced age, previous hospital treatment, and the presence of one or more underlying diseases were associated with critical illness and death by COVID‐19.

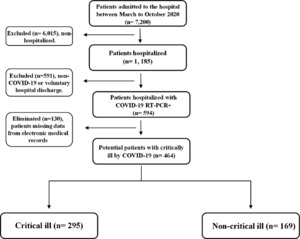

## 1 Introduction

In mid‐December 2019, a new respiratory virus was identified in Wuhan, China. This virus caused unexplained pneumonia and was named severe acute respiratory syndrome coronavirus 2 (SARS‐CoV‐2), responsible for the coronavirus disease 2019 (COVID‐19) [1]. This virus has spread worldwide, with 774 631 444 cases and 7 031 216 deaths reported; in Mexico, 7 700 000 cases and more than 335 000 deaths have been reported (accessed 29 February 2024) [2].

COVID‐19 is one of the most important diseases in recent years; this syndrome is characterized by fever, dry cough, and dyspnea, among other symptoms [3]. The clinical severity of COVID‐19 has been classified as mild, moderate, or severe depending on various factors [4]. Some cases may progress from severe to critical; the difference between the two states of COVID‐19 is that in critical patients, there is respiratory failure requiring mechanical ventilation, shock, and organ failure, which require intensive care unit (ICU) admission [5]. Approximately 5%–14% of COVID‐19 cases develop into critical illness [6]. Studies in different countries have associated risk factors with critical COVID‐19 illness, such as demographic factors (e.g., age or sex), symptoms (e.g., fever or dyspnea), underlying diseases (e.g., obesity or hypertension), and laboratory parameters (e.g., C‐reactive protein or D‐dimer) [7]. The critical status of COVID‐19 is a condition highly predictive of patient death; in fact, critically ill patients have a higher mortality rate of around 61.5% [8].

There are reports in other countries where patient characteristics are associated with the severity of COVID‐19; in China, symptoms such as fever, cough, myalgia, cough, lymphopenia, and lung lesions were associated with severe COVID‐19 [9, 10, 11]. In European countries, headache, loss of smell, nasal obstruction, and cough, among others, are key symptoms to progress from mild to severe COVID‐19 [12]. Furthermore, in Bulgaria, the levels of white blood cells, C‐reactive protein, creatinine, aspartate aminotransferase, lactate dehydrogenase, ferritin, fibrinogen, and D‐dimer were associated with severe cases of COVID‐19 [13].

Previous studies (2021) suggested a 41% incidence of SARS‐CoV‐2 in 26 Mexican states [14]. Concerning the west of Mexico, the authors reported a prevalence of 29.8%; in the northwest, the prevalence was 45.2%, and it was 42.7% in the south of Mexico [15, 16]. Unfortunately, most of these studies considered mild COVID‐19 cases, and the data on the risk factors associated with COVID‐19 and critically ill patients in Mexico are limited. Therefore, our objective was to associate the demographic, clinical, symptomatic, underlying disease, and laboratory parameters of patients with COVID‐19 with critical illness and death in northwestern Mexico.

## 2 Material and Methods

### 2.1 Case Definition

This work was a comparative cross‐sectional study in which critically ill patients from the Hospital General Regional No. 1 of the Instituto Mexicano del Seguro Social, located in the state of Sinaloa, in northwestern Mexico, were included. According to the National Institute of Health (NIH) [17], a critically ill patient was defined as one who had respiratory failure, septic shock, and/or multiple organ dysfunction.

### 2.2 Inclusion and Exclusion Criteria

All patients older than 18 who were hospitalized at the Hospital General Regional No. 1 with COVID‐19, diagnosed by RT‐PCR testing, were included. The hospitalized patients with suspected COVID‐19 but without RT‐PCR testing and the hospitalized patients with COVID‐19 diagnosed by RT‐PCR testing who requested voluntary hospital discharge were excluded. The hospitalized patients with COVID‐19 diagnosed by RT‐PCR testing but whose electronic medical records were missing demographic, clinical, or laboratory data were also excluded.

### 2.3 Data Collection and Participants

The patients' electronic medical records were collected from March to October 2020. For this study, a representative sample of hospitalized patients who were critically ill with COVID‐19 was selected using the ratio formula *Z*α 2 (*p*)(*q*)/*d*2, based on the prevalence of patients hospitalized with COVID‐19 in Mexico, and a minimum of 384 participants was needed. We collected 1185 electronic medical records, of which 591 were excluded. Figure S1 shows the steps for subject selection.

Sociodemographic and clinical characteristics, such as sex, age, occupation, demographics, education, and history of treatment for COVID‐19 prior to hospitalization, were collected from the electronic medical records. The symptoms included fever, cough, chest pain, dyspnea, headache, irritability, diarrhea, vomiting, chills, abdominal pain, myalgia, arthralgia, malaise, polypnea, sore throat, conjunctivitis, and cyanosis. The underlying diseases included diabetes mellitus, chronic obstructive pulmonary disease (COPD), asthma, immunosuppression, arterial hypertension, cardiovascular disease, obesity, chronic renal failure, and chronic hepatic failure. The vital signs included were temperature, heart rate, respiratory rate, and blood pressure.

### 2.4 RT‐PCR Assay

To identify SARS‐CoV‐2 from throat swab samples of patients, RNA extractions and RT‐PCR were performed on all samples following protocols and guidelines for laboratory surveillance of respiratory viruses of the Institute of Epidemiological Diagnosis and Reference (Instituto de Diagnóstico y Referencia Epidemiológicos, InDRE) [18].

### 2.5 Laboratory Tests

The laboratory tests taken were hemoglobin, leukocytes, leukocyte differential, platelets, glucose, creatinine, albumin, total protein, D‐dimer, and C‐reactive protein, which were taken following the procedures of Hospital General Regional No. 1 of the Instituto Mexicano del Seguro Social, using the VITROS XT 7600 Integrated Systems (Ortho Clinical Diagnostic, New Jersey, United States).

### 2.6 Imaging and Invasive Procedures

To the computed tomography chest, patients were prepared following the procedures of Hospital General Regional No. 1 of the Instituto Mexicano del Seguro Social; the computed tomography was made in the tomograph ingenuity core/IntelliSpace (Philips, Netherlands, Amsterdam) with 64 cuts.

### 2.7 Statistical Analysis

The measures of central tendency, mean, mode, median, dispersion with standard deviation, and variance were used for the quantitative variables, and percentages and frequencies were used for the qualitative variables. The *X*‐squared test was used to compare the qualitative variables. For qualitative variables, the Student's *t*‐test, or the Mann–Whitney *U* test, was used. Multivariate analyses with logistic regression, odds ratio, and 95% confidence intervals were used to determine associations. A *p* value ≤ 0.05 was considered statistically significant. The data were analyzed using SPSS® Statistics Version 24 (IBM Corp., Armonk, NY, United States).

### 2.8 Ethical Consideration

The present study was approved by the Ethics Committee of the Women's Hospital, Secretariat of Health (No. 202302–14), and the principles of the Declaration of Helsinki of the World Medical Association were followed, generally using a waiver of consent, given the need to only collect routinely available clinical data, with no need for additional study‐specific diagnostic testing.

## 3 Results

### 3.1 Characteristics of the Study Population

In total, 594 patients with COVID‐19 were included in this study, of whom 57.9% (344/594) were male and 42.1% (250/594) were female; most were unemployed (67%, 398/594) and urban (86.7%, 515/594) and had a primary education (51.2%, 304/594), as shown in Table 1. In addition, 52.7% (313/594) of the patients had received previous treatment for COVID‐19. The patients with COVID‐19 also had various underlying diseases, the most frequent being hypertension at 55.4% (329/594), followed by T2D at 34.5% (205/594), obesity at 23.2% (138/594), chronic kidney disease at 7.2% (43/594), and COPD at 5.1% (30/594); there were lesser proportions of acute myocardial infarction, asthma, dyslipidemia, immunosuppression, and chronic liver failure, as shown in Table 1.

**TABLE 1:** Sociodemographic and clinical characteristics of the COVID‐19 patients.

Characteristics	Total *n* = 594	%
**Sex**		
Male	344	57.9
Female	250	42.1
**Occupation**		
Unemployed	398	67.0
Employed	196	33.0
**Demography**		
Urban	515	86.7
Rural	79	13.3
**Education**		
Illiterate	33	5.6
Elementary	304	51.2
High school	54	9.1
University	72	12.1
**Previous treatment**	313	52.7
**Underlying disease**	458	77.1
Hypertension	329	55.4
Type 2 diabetes	205	34.5
Obesity	138	23.2
Chronic kidney disease	43	7.2
COPD	30	5.1
Acute myocardial infarction	17	2.9
Asthma	13	2.2
Dyslipidemia	13	2.2
Immunosuppression	12	2.0
Chronic liver failure	2	0.3

Abbreviation: COPD, chronic obstructive pulmonary disease.

### 3.2 Characteristics of the Critically Ill COVID‐19 Patients

**FIGURE 1:**
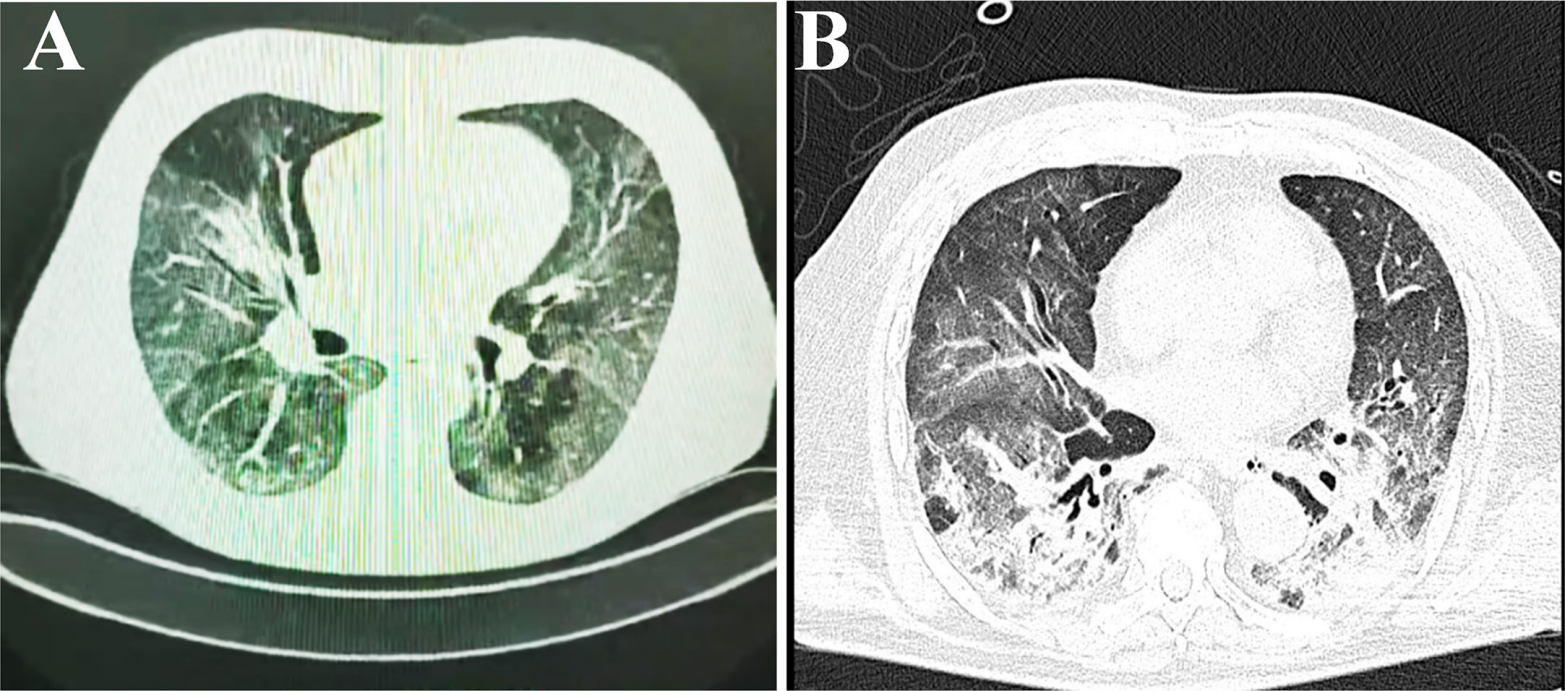
Chest computed tomography scan of two patients with critical illness due to COVID‐19. (A) A 58‐year‐old male, RT‐PCR positive for SARS‐CoV‐2, has a chest tomography scan with multiple ground‐glass lesions, reticulations, and condensation areas, CO‐RADS 6 classifications. (B) A 47‐year‐old male, RT‐PCR positive for SARS‐CoV‐2, has a chest tomography scan with ground‐glass opacities, areas of atelectasis, and bilateral parenchymal lesions, CO‐RADS 6 classifications.

Of the total number of patients (594), 130 could not be diagnosed as critically ill due to insufficient information in the clinical history (clinical or biochemical parameters), but we know that 53% (69/130) of them were male, 47% (61/130) were female, and 46% (60/160) died, leaving a total of 464 patients, of whom 64% (295/464) became critically ill because all these patients presented respiratory failure (Figure 1 shown chest computed tomography of two patients with critical ill with pulmonary damage), and dysfunction of two or more organs, and 36% (169/464) did not, as shown in Table 2. Some sociodemographic, clinical, and baseline disease characteristics were associated with patients who were critically ill with COVID‐19. Regarding sex, no statistical differences were found between men and women. On the other hand, the mean age of the COVID‐19 patients who became critically ill was higher than that of the noncritical patients (66 vs. 61 years, *p*: 0.003). Younger patients were associated with noncritical (< 49:14.2% vs. 23.1%, *p*: 0.02) COVID‐19, and the age range of 50–59 also had more noncritical cases; however, the patients aged 60–67 years were similar in both study groups, and the age groups of 68–75 and > 75 had a higher number of critically ill than noncritically ill patients, but the difference was not statistically significant (Table 2). Another sociodemographic characteristic associated with critical illness was occupation, as unemployed patients were more frequently critically ill than noncritically ill (72.9% vs. 56.2%, *p*: <0.0001). No statistical differences were found between the demographic characteristics of the critically ill patients (Table 2).

**TABLE 2:** Sociodemographic and clinical characteristics and underlying diseases related to critically ill COVID‐19 patients.

The characteristics	Total	Critically ill (%)	Noncritically ill (%)	*p* value
	*n* = 464 (%)	*n* = 295 (64.0)	*n* = 169 (36.0)	
**Sex**				
Male	275 (59.3)	167 (56.6)	108 (63.9)	0.124
Female	189 (40.7)	128 (43.4)	61 (36.1)	
**Age**				
Median (IQR)	64.5 (54–73)	66 (55–74)	61 (51–70)	0.003*
< 49	81 (17.5)	42 (14.2)	39 (23.1)	0.02*
50–59	95 (20.5)	55 (18.6)	40 (23.7)	0.18
60–67	99 (21.3)	63 (21.4)	36 (21.3)	0.9
68–75	96 (20.7)	68 (23.1)	28 (16.6)	0.12
> 75	93 (20)	67 (22.7)	26 (15.4)	0.07
**Occupation**				
Unemployed	310 (66.8)	215 (72.9)	95 (56.2)	< 0.0001*
Employed	154 (33.2)	80 (27.1)	74 (43.8)	
**Demography**				
Urban	393 (84.7)	241 (81.7)	152 (89.9)	0.018
Rural	71 (15.3)	54 (18.3)	17 (10.1)	
**Before critical illness**				
Days before critical illness (IQR)	7 (4–10)	7 (3–10)	7 (4–10)	0.48
Pharmacological treatment before critical illness	265 (57.1)	176 (59.7)	89 (52.7)	0.143
Steroid treatment before critical illness	167 (36.0)	153 (51.9)	14 (8.3)	< 0.0001*
Antimicrobial treatment before critical illness	152 (32.8)	132 (44.7)	20 (11.8)	< 0.0001*
**Underlying diseases**	360 (77.6)	240 (81.4)	120 (71.0)	0.01*
Hypertension	262 (56.5)	177 (60.0)	85 (50.3)	0.042*
Type 2 diabetes	155 (33.4)	94 (31.9)	61 (36.1)	0.353
Obesity	109 (76.5)	80 (72.9)	29 (82.8)	0.015*
Chronic kidney disease	32 (6.9)	26 (8.8)	6 (3.6)	0.031*
COPD	27 (5.8)	16 (5.4)	11 (6.5)	0.631
Acute myocardial infarction	14 (3.0)	7 (2.4)	7 (4.1)	0.284
Dyslipidemia	11 (2.4)	6 (2.0)	5 (3.0)	0.529
Asthma	9 (1.9)	5 (1.7)	4 (2.4)	0.614
Immunosuppression	9 (1.9)	6 (2.0)	3 (1.8)	0.846
**Number of underlying diseases**				
0	124 (26.7)	69 (23.4)	55 (32.5)	0.028*
1	131 (28.2)	86 (29.2)	45 (26.6)	0.59
2–3	187 (40.3)	126 (42.7)	61 (36.1)	0.16
≥ 4	22 (4.7)	14 (4.7)	8 (4.7)	0.99

*Note:* The chi square test was used to obtain the statistical significance, and the Mann–Whitney *U* test was applied to obtain the statistical significance in the variables by IQR.

Abbreviations: COPD, chronic obstructive pulmonary disease; IQR, interquartile range.

* *p* value ≤ 0.05.

In addition, some of the patients' data before their critical illness with COVID‐19 (e.g., days of COVID‐19 and pharmacological treatment) were analyzed for associations with critical illness, as shown in Table 2. No statistical difference was found between days with COVID‐19 and overall pharmacological treatment with critical illness. At the same time, previous steroid use (51.9% vs. 8.3%, *p*: < 0.0001) and antibiotics (44.7% vs. 11.8%, *p*: < 0.0001) were associated with critical illness, as compared to noncritical illness (Table 2).

Regarding the underlying diseases, the presence of underlying diseases was associated with critical illness (81.4% vs. 71%, *p*: 0.01), as compared to noncritical illness (Table 2). Among all the previously mentioned diseases (Table 1), hypertension (60% vs. 50.3%, *p*: 0.04), obesity (72.9% vs. 17.1%, *p*: 0.015), and chronic kidney disease (8.8% vs. 3.6%, *p*: 0.031) were associated with critical illness. In addition, the number of underlying diseases in patients with COVID‐19 was analyzed; having zero underlying diseases was associated with noncritically ill patients (23.4% vs. 32.5%, *p*: 0.028); the prevalence of patients with one (29.9% vs. 26.6%) and one to three (42.7% vs. 36.1%) underlying diseases was higher in critically ill patients, but no statistical differences were found. The distribution of patients with four or more underlying diseases was similar between the critically ill and noncritically ill patients (Table 2).

### 3.3 Association of Symptoms of COVID‐19 and Critical Illness

Several symptoms were reported in patients with COVID‐19, the most common being dyspnea (83.1%), followed by fever and cough (77.1% each), headache (62.5%), myalgia (55.6%), arthralgia (51.2%), and chest pain (36.4%), with smaller proportions of odynophagia (28.6%), chills (21.1%), rhinorrhea (17.2%), and abdominal pain (12.2%), among others, as shown in Table 3. As for the distribution of symptoms in patients with critical illness and noncritical illness, it was similar in both study groups, so no statistical differences were found between COVID‐19 symptoms and critical illness, as shown in Table 3.

**TABLE 3:** Symptoms associated with critically ill COVID‐19 patients.

Symptoms	Total	Critically ill (%)	Noncritically ill (%)	*p* value
	*n* = 464 (%)	*n* = 295 (64.0)	*n* = 169 (36.0)	
Dyspnea	386 (83.1)	241 (81.7)	145 (81.7)	0.25
Fever	358 (77.1)	228 (77.3)	130 (76.9)	0.92
Cough	358 (77.1)	230 (78)	128 (75.7)	0.58
Headache	290 (62.5)	184 (62.4)	106 (62.7)	0.94
Myalgia	258 (55.6)	167 (56.6)	91 (53.8)	0.56
Arthralgia	238 (51.2)	151 (51.2)	87 (51.5)	0.95
Thoracic pain	169 (36.4)	115 (39.0)	54 (32)	0.13
Odynophagia	133 (28.6)	86 (29.2)	47 (27.8)	0.75
Chills	98 (21.1)	63 (21.4)	35 (20.7)	0.87
Rhinorrhea	80 (17.2)	53 (18)	27 (16.0)	0.58
Abdominal pain	57 (12.2)	40 (13.6)	17 (10.5)	0.26
Anosmia	32 (6.8)	20 (6.8)	12 (7.1)	0.89
Polypnea	31 (6.6)	15 (5.1)	16 (9.5)	0.06
Dysgeusia	27 (5.8)	17 (5.8)	10 (5.9)	0.94
Cyanosis	13 (2.8)	10 (3.4)	3 (1.8)	0.31
Nasal congestion	2 (0.4)	0 (0.0)	2 (1.2)	0.13

*Note:* The chi square test was used to obtain statistical significance.

### 3.4 The Clinical and Laboratory Findings of COVID‐19 in Critically Ill Patients

The clinical and laboratory data of patients with COVID‐19 are shown in Table 4. Some of these data were related to patients who developed critical illness; for example, the heart rate (97 vs. 90 beats/min, *p*: 0.014), respiratory rate (24 vs. 22 breaths/min, *p*: 0.002), glucose (143 vs. 130.34 mg/dL, *p*: 0.033), creatinine (1 vs. 0.8 mg/dL, *p*: < 0.001), albumin (3.3 vs. 0.8 g/dL, *p*: < 0.001), leukocytes (12.4 vs. 10.6 count/μL, *p*: 0.018), neutrophils (10.8 vs. 8.8 count/μL, *p*: 0.004), leukocyte index (14.29 vs. 10.57, *p*: < 0.001), D‐dimer (2100 vs. 647 ng/mL, *p*: < 0.001), and C‐reactive protein (15.9 vs. 12.35 mg/L, *p*: 0.008) were all higher in critically ill patients compared to noncritically ill patients, as shown in Table 4. On the other hand, the oxygen saturation (83 vs. 89%, *p*: < 0.001), diastolic pressure (76 vs. 78 mm/Hg, *p*: 0.036), hemoglobin (13 vs. 14, *p*: < 0.001), lymphocytes (0.7 vs. 0.8 count/μL), and platelets (236 vs. 269 count/μL, *p*: <0.001) were lower in the critically ill patients than the noncritically ill patients, as shown in Table 4. The distribution of the rest of the data was similar in both study groups.

**TABLE 4:** Vital signs, symptoms, and laboratory values that are associated with critically ill COVID‐19 patients.

Clinical vital signs and laboratory parameters	Critically ill	Noncritically ill	*p* value
	*n* = 295 (IQR)	*n* = 169 (IQR)	
Temperature (°C)	37 (36.6–37.6)	37 (36.7–37.6)	0.848
Heart rate (beats per minute)	97 (84–108)	90 (79–107)	0.014*
Breathing frequency (breath per minute)	24 (21–28)	22 (20–25)	0.002*
Oxygen saturation (%)	83 (68–89)	89 (84–93)	< 0.001*
Systolic pressure (mm/Hg)	125 (110–142)	126 (116–142)	0.372
Diastolic pressure (mm/Hg)	76 (66–82)	78 (71–84)	0.036*
Glucose (mg/dL)	143 (104–218.9)	130.34 (91–199.48)	0.033*
Creatinine (mg/dL)	1 (0.8–1.6)	0.8 (0.7–1.1)	< 0.001*
Albumin (g/dL)	3.3 (2.9–3.7)	0.8 (3.2–3.9)	< 0.001*
Hemoglobin (g/dL)	13 (11.5–14.3)	14 (12.7–15)	< 0.001*
Hematocrit (%)	38 (38–38)	38 (38–38)	0.717
Leucocyte count/μL	12.4 (8.5–16.7)	10.6 (8.2–14.2)	0.018*
Neutrophil count/μL	10.8 (7.1–14.8)	8.8 (6.2–12.3)	0.004*
Lymphocyte count/μL	0.7 (0.5–1)	0.8 (0.6–1.2)	< 0.001*
leukocyte index	14.29 (8.56–26.33)	10.57 (6.5–17.38)	< 0.001*
Platelet count/μL	236 (172–324)	269 (223–335)	< 0.001*
D‐Dimer (ng/mL)	2100 (614–6657)	647 (320–1531)	< 0.001*
C‐reactive protein (mg/L)	15.9 (9–23.62)	12.35 (7.2–21)	0.008*

*Note:* The Mann–Whitney *U* test was applied to obtain the statistical significance.

Abbreviations: μL, microliters; dL, deciliters; Hg, mercury, grams; IQR, interquartile range; mg, milligrams.

* *p* value ≤ 0.05.

### 3.5 The Characteristics of Patients With COVID‐19 Associated With Critical Illness and Death

To find associations with critical illness or death, the factors most representative of COVID‐19 patients in the bivariate analyses were subjected to multivariate analysis (logistic regression), as shown in Table 5. Regarding critical illness, associations were found between the age of patients and critical illness; comparisons were made between each of the different age groups versus < 49 years (which were the youngest patients with critical illness), of which 60–67 years (OR: 2.03, CI: 1.2–3.5, *p*: 0.004), 68–75 years (OR: 2.55, CI: 1.5–4.4, *p*: 0.0002), and > 76 years (OR: 2.67, CI: 1.6–4.6, *p*: 0.0002) were associated with critical illness, as shown in Table 5. In addition, the use of treatments before being critically ill was associated with this condition (OR: 1.61, CI: 1.1–2.3, *p*: 0.023), and the presence of an underlying disease (OR: 1.73, CI: 1.1–2.7, *p*: 0.015) and > 2 underlying diseases (OR: 1.9, CI: 1.3–3.02, *p*: 0.001) versus 0 underlying diseases was associated with being critically ill, as shown in Table 5. No association was found between sex or demographics and critical illness.

**TABLE 5:** Demographic and clinical factors associated with critical illness and death from COVID‐19.

Characteristics	Critical illness *n* = 295			Death *n* = 321		
	OR	(95% CI)	*p* value	OR	(95% CI)	*p* value
**Sex**						
Female vs. male	1.06	(0.7–1.5)	0.77	0.94	(0.6–1.3)	0.68
**Demography**						
Rural vs. urban	1.05	(0.6–1.7)	0.91	1.21	(0.7–1.9)	0.55
**Age**						
50–59 vs. < 49	1.45	(0.9–2.5)	0.1	1.7	(1.0–2.9)	0.023*
60–67 vs. < 49	2.03	(1.2–3.5)	0.004*	2.8	(1.6–4.4)	< 0.0001*
68–75 vs. < 49	2.55	(1.5–4.4)	0.0002*	4.5	(2.5–7.2)	< 0.0001*
> 76 vs. < 49	2.67	(1.6–4.6)	0.0002*	6.68	(3.7–12.5)	< 0.0001*
**Previous treatment**						
Yes vs. no	1.61	(1.1–2.3)	0.023*	1.54	(1.1–2.2)	0.02*
**Underlying diseases**						
1 vs. 0	1.73	(1.11–2.7)	0.015*	1.8	(1.2–2.9)	0.0057*
> 2 vs. 0	1.9	(1.3–3.02)	0.001*	2.4	(1.5–3.6)	< 0.0001*

*Note:* Logistic regression was applied to obtain the statistical significance.

Abbreviations: CI: confidence index; OR: odds ratio.

* *p* value ≤ 0.05.

On the other hand, we also looked for associations between patients with COVID‐19 and death; as with the critically ill patients, some factors were associated with death, as shown in Table 5. All age groups of the patients analyzed with COVID‐19 were associated with death: 50–59 years (OR: 1.7; CI: 1.0–2.9; *p*: 0.023), 60–67 years (OR: 2.8; CI: 1.6–4.4; *p*: < 0.0001), 68–78 years (OR: 4.5; CI: 2.5–7.2; *p*: < 0.0001), and > 76 years (OR: 6.68; CI: 3.7–12.5; *p*: < 0.0001). In addition, the use of previous treatments (OR: 1.54; CI: 1.1–2.2; *p*: 0.02), as well as 1 (OR: 1.8; CI: 1.2–2.9; *p*: 0.0057) and > 2 (OR: 2.4; CI: 1.5–3.6; *p*: < 0.0001) underlying diseases, were associated with death in COVID‐19 patients, as shown in Table 5.

In addition, we analyzed the interaction between variables in the model, considering gender as the focal independent variable. The results show that no statistical differences were generated in the variables analyzed. Still, we should consider that as more than two variables are involved, the parsimony of the model (the principle of using the simplest and most concise model that adequately represents the data) may be affected. The results may not be significant (Table S1).

## 4 Discussion

COVID‐19 is a respiratory disease that has caused many deaths worldwide, and Mexico has been one of the most affected countries. The patient response to COVID‐19 is key to the patient's survival of this disease, as various patient factors have been associated with COVID‐19 severity, hospitalization for critical illness, and death. In this work, we demonstrated that in adult subjects from northwestern Mexico (Sinaloa State), some sociodemographic (e.g., age or occupation), clinical (e.g., previous steroid or antibiotic use), and underlying disease (e.g., hypertension or obesity) characteristics were more prevalent in patients who were critically ill with COVID‐19. No COVID‐19 symptoms were associated with the critically ill patients. In addition, some vital signs and laboratory parameters (e.g., heart rate, oxygen saturation, glucose, leukocyte index, and D‐dimer, among others) were associated with critically ill patients. Finally, age, previous treatment, and underlying diseases were associated (by logistic regression) with critically ill patients as well as with death from COVID‐19.

In the present study, the proportion of men and women with critical illness was similar; this result differed from other studies because the proportion of men critically ill with COVID‐19 is higher than that of women [1, 19, 20]. This phenomenon could be related to the fact that in Mexico, some underlying diseases associated with critical illness in COVID‐19, such as obesity and hypertension, are more prevalent in adult women (75% and 64.2%, respectively) than in men (69.6% and 49.9%, respectively) [21]. In terms of age, the patients with critical illness were older than the patients without critical illness, with a median age of 66 years versus 61 years in this study. These data were consistent with several studies, most of which found patients with critical illness to be older than 63 years [22, 23, 24].

One of the characteristics of patients with COVID‐19 who became critically ill was that they were using medications such as steroids and antibiotics at the onset of the disease. The use of corticosteroids in COVID‐19 was a controversial decision. Although several studies showed that using corticosteroids in mild or moderate cases of COVID‐19 did not reduce the mortality, length of hospital stay, or duration of viral shedding, Mexican personnel continued to use them [25]. The use of corticosteroids has been associated with an increased risk of death or secondary infection during hospitalization [26]. The use of corticosteroids in noncritically ill patients could inhibit the protective function of T cells and affect the B cells in the production of antibodies, which could lead to an increase in plasma viral load; it could also block macrophages from attacking secondary nosocomial infections, and all these phenomena could allow the patient to progress from noncritically ill to critically ill [27]. Regarding the use of antibiotics, most cases of COVID‐19 did not present a bacterial coinfection that would justify the use of antibiotics, and the indiscriminate use of antibiotics could affect the health of patients with COVID‐19; in fact, a study by Bendala Estrada et al. found higher mortality rates in patients with COVID‐19 who used antibiotics than in those who did not use antibiotics [28]. Indiscriminate use of antibiotics in patients with COVID‐19 increased antimicrobial resistance and the likelihood of developing coinfection with multidrug‐resistant or extensively resistant bacteria [29]. On the other hand, we cannot rule out the fact that the naturally poor evolution of COVID‐19 led patients to use steroids or antibiotics. The treatments for mild COVID‐19 patients are a controversial issue that needs further research. In addition, more training is needed for medical staff in mild COVID‐19 cases.

Other important characteristics associated with critically ill patients were underlying diseases such as hypertension, obesity, or chronic kidney disease; these data were consistent with other reports. Wang et al. observed that the prevalence of hypertension was higher in ICU patients than in non‐ICU patients (58.3% vs. 21.6%; *p* < 0.001) [30]. Similarly, Hernández‐Cárdenas et al. showed that obesity was the most frequent underlying condition in patients with COVID‐19 who died [31]. In addition, Ng et al. [32] found that patients with COVID‐19 and chronic kidney disease were more likely to be hospitalized than those without COVID‐19 (31.7% vs. 25.4%). On the other hand, several studies related the presence of T2D with critically ill patients in COVID‐19 [33, 34, 35], but contradictorily, we found no relationship between patients with T2D and critically ill patients; in fact, the distribution of these patients was similar between the critically ill and noncritically ill patients. This could be since in Mexico, the prevalence of T2D in subjects older than 20 years is very high (31.2%), a fact that could affect the distribution of patients with T2D [21].

Regarding COVID‐19 symptoms, the most common symptoms found in this study were consistent with other studies [19, 31, 36]. However, although some symptoms were associated with critically ill patients, such as fever, dyspnea, nausea, vomiting, or diarrhea [37, 38], the distribution of COVID‐19 symptoms in this study was similar between the two study groups. In addition, this study's vital signs and laboratory parameters related to critically ill patients were consistent with other works [7]. Liang et al. found that creatinine, C‐reactive protein, and neutrophil levels were higher in critically ill patients than noncritically ill patients, while leukocytes, platelets, and hemoglobin were lower in critically ill patients [39]. Alharthy et al. found that heart rate, respiratory rate, and D‐dimer levels, among other parameters, were higher in critically ill patients who died compared to those who did not die [36]. The search for biomarkers (symptoms, vital signs, or laboratory parameters) to identify critical cases in COVID‐19 patients should continue; in this sense, we could improve the early identification of these patients and treatment to prevent death in COVID‐19 patients.

Finally, logistic regression was used to identify the association between the demographic and clinical COVID‐19 characteristics with critical illness or death. We found associations such as age, use of previous treatments, and underlying diseases using this statistical tool. Interestingly, the older the age of patients with COVID‐19, the higher the probability of developing critical illness or death. This phenomenon was also observed for underlying diseases (hypertension, obesity, CKD, and T2D, among others), and the more underlying diseases that the COVID‐19 patients had, the higher the probability of developing critical illness or death. These data were in line with other studies. Li et al. (2020) showed that patients with COVID‐19 ≥ 65 years were 2.25 times more likely to become critically ill than those ≤ 65 years (*p*: 0.000) [40]. Wu et al. also found that symptomatic COVID‐19 patients ≥ 59 years were 5.1 times more likely to die than those ≤ 30 years [41]. Martínez‐Martínez et al. showed that patients of older age were associated with severe COVID‐19 compared to those nonsevere (58.2 vs. 40.1 years, *p*: < 0.001) in a study of Mexican subjects. In addition, they showed that age (younger or older) could modify some risk factors associated with severe COVID‐19, such as COPD, asthma, and immunosuppression, among others [42]. The increased association between age and critical illness in COVID‐19 may be due to older subjects having more underlying diseases, a weakened immune system, higher proinflammatory cytokines, possibly lower ACE2 levels, and an increased SARS‐CoV‐2 viral load [7]. Regarding major underlying diseases, there is a strong association between critical illness and some underlying diseases; for example, Huang et al. showed that hypertension increased the odds of the severity of and death from COVID‐19 by 1.5 and 1.2 times, respectively [43]. Vera‐Zertuche et al. showed that obesity alone increased the risk of death 2.7‐fold, but obesity with other comorbidities increased the risk up to 5.06‐fold [44]. Guo et al. showed that patients with T2D had a 2.96‐fold increased risk of severe COVID‐19 or death [34]. Docherty et al. showed that patients with CKD had a 1.19–1.39‐fold increased risk of death [45].

The strong association between metabolic diseases and critical illness could be due to the physiological changes induced by these diseases; for example, hypertension alters the balance of the renin‐angiotensin‐aldosterone system pathway, downregulating the angiotensin‐converting enzyme (ACE) 2 and increasing the ACE/angiotensin II, facts that favor colonization and infection by SARS‐CoV‐2 [46]. In addition, patients with hypertension tend to be older individuals with other comorbidities such as obesity or T2D [47]. Patients with obesity have altered immune system function, chronic inflammation, ACE2 highly expressed in adipose tissue, low vitamin D concentration, and overexpression of molecules associated with SARS‐CoV‐2 infection such as MCT4, alpha integrin, and activated T cell nuclear factor 1, among others [48, 49]. In T2D, patients with this metabolic disease tend to be older and have other comorbidities such as obesity or hypertension and overexpression of ACE2, inflammation, and endothelial activation related to insulin resistance [50, 51, 52].

On the other hand, the use of the markers shown in this study, together with predicting mortality scores, could help to avoid critical illness or death from COVID‐19. Alanís‐Naranjo et al. compared two predictors of mortality in Mexican hospitalized COVID‐19 patients: the pneumonia severity index (PSI) or PORT score (PORT/PSI) and the Sequential Organ Failure Assessment (SOFA) score. The PORT/PSI score was better than the SOFA in predicting deaths in hospitalized Mexican subjects with COVID‐19 [53].

Fortunately, we now have several COVID‐19 vaccines that can protect against severe SARS‐CoV‐2 infection; in fact, the use of COVID‐19 vaccines has greatly reduced severe cases and deaths from COVID‐19 worldwide. However, there is still a significant percentage of the population that has not received one or more doses. Approximately 70.9% (5.44 billion) of the world's population has received a dose of COVID‐19 vaccine, but 29.1% have not received a dose (2.3 billion) (accessed 11 November 2022) [54]. As for Mexico, 76% of the population has one dose, 64% has two doses, 45% has an additional dose, and 24% has no dose (accessed 11 November 2022), which means that more than 31 million Mexicans have not been vaccinated [54]. There are still many people who, without vaccination, may develop critical illness due to COVID‐19, so it is necessary to continue the search for factors related to this condition or death from COVID‐19 to treat patients as effectively as possible. On the other hand, the levels of antibodies that vaccinated subjects with metabolic diseases, for example, obesity, hypertension, or diabetes, produce against SARS‐CoV‐2 are lower than subjects without metabolic diseases; considering that Mexico is a country with a high prevalence of these diseases, Mexican subjects with metabolic diseases vaccinated with one or two doses may still have an increased probability of suffering critical illness from COVID‐19 compared to those without metabolic diseases. However, further research on this topic is needed [55].

To our knowledge, this is the first study to identify the main epidemiologic and clinical characteristics and laboratory parameters associated with critical illness and death in patients hospitalized for COVID‐19 in northwestern Mexico. However, the limitations of this work were the sample size and the retrospective nature of the data; many patients were excluded from the study because they did not meet the inclusion criteria. In addition, the information in this work predated the COVID‐19 vaccine era.

## 5 Conclusion

In this study, some demographic (e.g., age and occupation) and clinical factors (e.g., previous treatment, underlying diseases, vital signs, and laboratory parameters) were associated with critical illness in patients hospitalized with COVID‐19 in northwestern Mexico. In addition, age (elderly patients), previous medication use, and the presence of underlying diseases were strongly associated with critical illness and death due to COVID‐19. The associations found in this work could be markers for Mexican subjects, which can help to avoid critical illness and death in COVID‐19 patients from the early stage of this disease.

## Author Contributions

Conceptualization: Francisco Martínez‐Villa, Uriel Angulo‐Zamudio, and Adrian Canizalez‐Roman. Data curation: Francisco Martínez‐Villa and Uriel Angulo‐Zamudio. Formal analysis: Ricardo González‐Esparza. Investigation: Nidia León‐Sicairos. Methodology: Nidia León‐Sicairos. Project administration: Adrian Canizalez‐Roman. Supervision: Hector Flores‐Villaseñor and Julio Medina‐Serrano. Visualization: Nidia León‐Sicairos. Writing–original draft: Francisco Martínez‐Villa, Uriel Angulo‐Zamudio, and Adrian Canizalez‐Roman. Writing–review and editing: Jaime Sanchez‐Cuen, Jesus Martinez‐Garcia, Hector Flores‐Villaseñor, and Julio Medina‐Serrano.

## Disclosure

Patients and/or the public were not involved in the design, conduct, reporting, or dissemination plans of this research. This submission has not been published anywhere previously, and it is not simultaneously being considered for publication by any other journal.

## Ethics Statement

The study was approved by the Ethics Committee of the Women’s Hospital, Secretariat of Health (No. 202302–14).

## Conflicts of Interest

The authors declare no conflicts of interest.

## Supporting information


**Table S1** Effect of variable interaction in associations with critical ill and death of COVID‐19 patients.

Figure S1

## Data Availability

The datasets generated and/or analyzed during the current study are available from the corresponding author on reasonable request.
