# Supplementary material for: Clinical Characteristics of Hospitalized Patients With COVID‐19 and Their Association With the Progression to Critical Illness and Death: A Single‐Center Retrospective Study From Northwestern Mexico
Source: Clin Respir J. 2024 Jul 16;18(7):e13813. doi: 10.1111/crj.13813 (PMC11251732; doi:10.1111/crj.13813)
Supplement: Supplementary file 1 — Table S1 Effect of variable interaction in associations with critical ill and death of COVID‐19 patients. [file CRJ-18-e13813-s002.docx]

| **Supplementary Table 1. Effect of variables interaction in associations with critical ill and death of COVID-19 patients.** | | | | | | | |
| --- | --- | --- | --- | --- | --- | --- | --- |
| **Interaction between varies** | **Critical ill n=295** | | |  | **Death n= 321** | | |
|  | **OR** | **(95% CI)** | **p value** |  | **OR** | **(95% CI)** | **p value** |
| Underlying diseases-demography-age 50-59, gender, and previous treatment | 1.1 | (0.32 – 3.7) | 0.88 |  | 1.05 | (0.3 – 3.59) | 0.93 |
| Underlying diseases-demography-age 60-67, gender and previous treatment | 1.75 | (0.69 – 4.44) | 0.23 |  | 1.76 | (0.68 – 4.5) | 0.23 |
| Underlying diseases-demography-age 68-75, gender, and previous treatment | 1.91 | (0.82-4.48) | 0.13 |  | 2.08 | (0.87 – 4.95) | 0.09 |
| Underlying diseases-demography-age >76, gender, and previous treatment | 2.42 | (0.9 – 6.46) | 0.07 |  | 2.32 | (0.72 – 7.2) | 0.16 |
| OR: odds ratio; CI: confidence index. Logistic regression was applied to get statistical significance. *: p-value ≤ 0.05 | | | | | | | |
