# Supplementary figures and images for: Clinical Characteristics of Hospitalized Patients With COVID‐19 and Their Association With the Progression to Critical Illness and Death: A Single‐Center Retrospective Study From Northwestern Mexico
Source: Clin Respir J. 2024 Jul 16;18(7):e13813. doi: 10.1111/crj.13813 (PMC11251732; doi:10.1111/crj.13813)

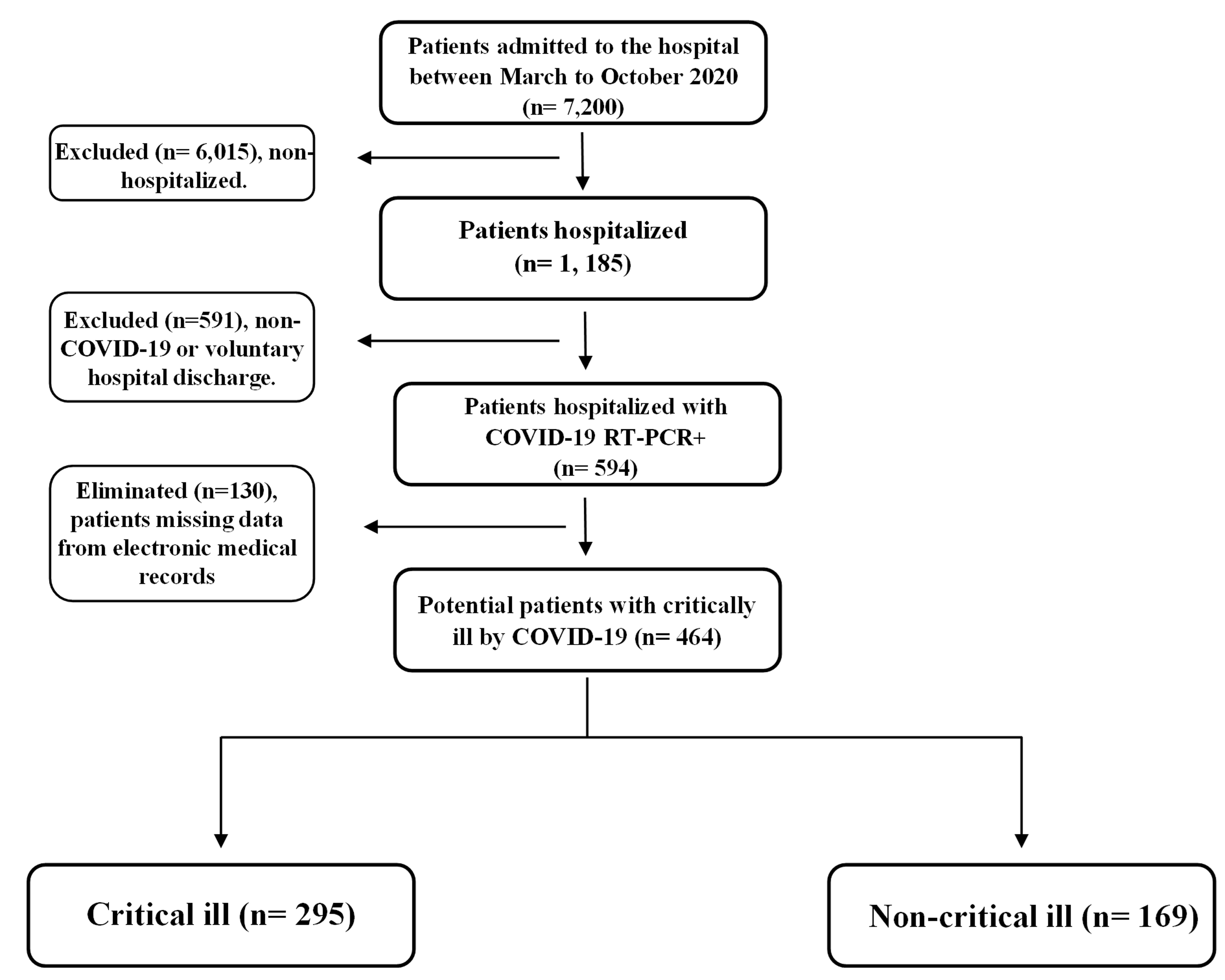

Supplement: Supplementary file 2 — Figure S1 [file CRJ-18-e13813-s001.tif]
